# Deployment of the consultation-liaison model in adult and child-adolescent psychiatry and its impact on improving mental health treatment

**DOI:** 10.1186/s12875-021-01437-5

**Published:** 2021-04-29

**Authors:** M.-J. Fleury, G. Grenier, L. Gentil, P. Roberge

**Affiliations:** 1 Department of Psychiatry, McGill University, Montreal, QC Canada; 2 Douglas Mental Health University Institute Research Center, Montréal, QC Canada; 3 Department of Family Medicine and Emergency Medicine, University of Sherbrooke, Sherbrooke, QC Canada

**Keywords:** Consultation-liaison model, Specialist respondent psychiatrists, Adult psychiatry, Child psychiatry, Adolescent psychiatry, Primary care, Barriers or facilitators, Model impacts

## Abstract

**Background:**

Little information exists on the perceptions of psychiatrists regarding the implementation and various impacts of the consultation-liaison model. This model has been used in Quebec (Canada) through the function of specialist respondent-psychiatrists (SRP) since 2009. This study assessed the main activities, barriers or facilitators, and impact of SRP in adult and child-adolescent psychiatry on the capacity of service providers in primary care and youth centers to treat patients with mental health disorders (MHD).

**Methods:**

Data included 126 self-administered questionnaires from SRP and semi-structured interviews from 48 SRP managers. Mixed methods were used, with qualitative findings from managers complementing the SRP survey. Comparative analyses of SRP responses in adult versus child-adolescent psychiatry were also conducted.

**Results:**

Psychiatrists dedicated a median 24.12 h/month to the SRP function, mainly involving case discussions with primary care teams or youth centers. They were confident about the level of support they provided and satisfied with their influence in clinical decision-making, but less satisfied with the support provided by their organizations. SRP evaluated their impacts on clinical practice as moderate, particularly among general practitioners (GP). SRP working in child-adolescent psychiatry were more comfortable, motivated, and positive about their overall performance and impact than in adult psychiatry. Organizational barriers (e.g. team instability) were most prevalent, followed by system-level factors (e.g. network size and complexity, lack of resources, model inflexibility) and individual factors (e.g. GP reluctance to treat patients with MHD). Organizational facilitators included support from family medicine group directors, collaboration with university family medicine groups and coordination by liaison nurses; at the system level, pre-existing relationships and working in the same institution; while individual-level facilitators included SRP personality and strong organizational support.

**Conclusion:**

Quebec SRP were implemented sparingly in family medicine groups and youth centers, while SRP viewed their overall impact as moderate. Results were more positive in child-adolescent psychiatry than in adult psychiatry. Increased support for the SRP function, adapting the model to GP in need of more direct support, and resolving key system issues may improve SRP effectiveness in terms of team stability, coordination among providers, access to MH services and readiness to implement innovations.

**Supplementary Information:**

The online version contains supplementary material available at 10.1186/s12875-021-01437-5.

## Background

Most individuals affected by mental health disorders (MHD) use primary care services as the point of entry to the mental health (MH) care system. Primary care is the gatekeepers to specialized MH services in Quebec (Canada), as in many countries [1, 2]. General practitioners (GP) are the main primary care providers, evaluating and treating in their clinics from 20 to 40% of patients with MHD annually [3–6]. Primary care services are less costly and stigmatizing, and more accessible than specialized MH services [7, 8]. Yet studies have reported limited capacity among primary care clinicians, especially GP, to diagnose and treat MH conditions, particularly severe or co-occurring MHD [5, 9–12] and substance-related disorders [13]. Better integration of specialized MH services into primary care has thus been strongly recommended and is at the heart of current international [14, 15] and Canadian [16] MH reforms.

Several models for integrating MH and primary care services have been developed in the past decades, including collaborative care [17], shared care [9] and the consultation-liaison model [18]. Collaborative and shared care originated with the Wagner chronic care model [19]. In shared care, psychiatrists work closely with GP [20], whereas collaborative care involves nurses, psychologists, social workers and other clinicians [21, 22], integrating three core work components: use of systematic psychiatric assessments; longitudinal patient monitoring and care management usually performed by nurses; and stepped-care recommendations by psychiatrists or other MH specialists [23]. Both collaborative and shared care may target specific populations (e.g. elderly, children and adolescents) and include clinician collaboration of varying intensity [22]. In the consultation-liaison model, psychiatrists hold regular case discussions with GP or other primary care clinicians, providing care management, clinician support and referrals without seeing patients directly [24–26]. The psychiatrist function is focused essentially on improving clinician skills [27]. Compared with the collaborative and shared care models, the consultation-liaison model implies fewer changes for primary care clinicians, as consultation-liaison essentially targets improvements to MH expertise, without reorganizing other areas of interdisciplinary group practice. Hybrid models also exist that blend characteristics of the three generic models [28, 29].

Randomized controlled trials have assessed the impacts of MH service integration in primary care on patient health outcomes [29–40] and determined that these models reduced psychiatric symptoms among patients with depression [29, 38, 39, 41–43], anxiety disorders [29, 40, 44], bipolar disorders [45] and multiple MHD [31]. Other studies focused on health service use found that integrated MH services increased access to MH services [20, 21] and reduced psychiatric hospitalizations [46, 47]. Other studies evaluated the impacts of MH service integration in primary care on clinical practice revealed improved MH screening [48–50] and prescribing practices [48].

Concerning the consultation-liaison model, patient outcome research found that the model improved MH conditions in the first 3 months of treatment while improvements in co-occurring MHD, alcohol and cannabis use appeared after 4 months [35]; depressive symptoms diminished as effectively with the consultation-liaison model as with collaborative care at 9-month follow-up [32]. Concerning heath service use, the consultation-liaison model improved treatment adherence and satisfaction with services between 3 and 12 months post-treatment [30], promoting efficiency in specialized MH service use [18, 27]. Finally, clinical practice improved in terms of GP knowledge and skills in diagnosing and treating patients with MHD [30, 51], medication prescribing [51], inter-professional communication [51, 52] and less stigmatizing attitudes [53].

Qualitative studies have assessed barriers and facilitators in implementing integrated MH services into primary care around multiple issues. At the system-level these included supportive legislative/policy environment [25, 54], availability of appropriate resources [55], preexisting relationships between specialized MH services and primary care [22, 56], and adequate incentives [25, 57–59]. Organizational-level issues included clinician acceptance of the model [60, 61], team stability [55], organizational support [1, 62, 63], leadership [55, 62], and feedback on model effectiveness [59]. Finally, individual-level concerns included interest among GP in MHD [1, 25, 59, 63], and individual qualities of psychiatrists [58, 60].

As implementation of the consultation-liaison model was not expected to involve sweeping changes, this model was selected in Quebec with the aim of improving MH expertise among providers in primary care, and youth centers which are specialized services treating patients under 18 years old, a majority of whom with MHD [64]. Moreover, this MH reform only aimed at changing practices in the public MH sector, which excludes the majority of GP who work in private practices under the direction of another Quebec ministry. The Quebec consultation-liaison model was based on the deployment of a specialist respondent-psychiatrist (SRP) function, launched as part of the 2005 Quebec MH Action Plan to help consolidate MH expertise outside of specialized psychiatric care [65]. Yet even the introduction of this “simplified” model of integrated specialized MH care was strongly contested by the Quebec Association of Psychiatric Physicians. An agreement was finally signed in 2009 between the Association and the Quebec government, establishing a high hourly fee for psychiatrists willing to serve as SRP in their health networks as a strong incentive for them to integrate this function into their professional activities. Under the 2009 agreement [66], one or two SRP were allocated either per 50,000 inhabitant in adult psychiatry or per 50,000 patients under age 18 in child-adolescent psychiatry. SRP had to be present on-site for 3.5 h/weekly to train clinicians working in MH primary care teams (MH-PCT), one-stop MH service teams, family medicine groups or youth centers, and provide on-call telephone availability during office hours between on-site visits.

MH-PCT and one-stop MH services were also mandated under the 2005 MH Action Plan [65]. MH-PCT located in local community health centers, the main public primary care organizations in Quebec, were projected to include 20 interdisciplinary psychosocial professionals and two GP. However, these original objectives have yet to be completed [67, 68]. One MH-PCT was allocated per 100,000 inhabitants among the health networks. MH-PCT offered individual and group therapies for patients with MHD. For networks with a minimum of 50,000 inhabitants, one-stop MH service teams were also established in local community health service centers as the point of entry for accessing MH services from either MH-PCT or psychiatric care. The one-stop MH service teams provide assessments for self-referred patients or those referred by GP, community organizations (voluntary sector) or by inter-sectorial resources (e.g. addiction rehabilitation centers). At the time they were created, one-stop MH service teams and MH-PCT were mainly staffed by MH professionals transferred from specialized MH services to primary care [67, 68]. Established in 2002 [69], family medicine groups brought together GP working in private practices and integrated secretarial support, as well as multidisciplinary teams that mainly included nurses and in some cases other psychosocial clinicians like social workers. Financed by the government, the enhanced family medicine groups ensured patient registration, and better continuity of care and access to care as they offered more days and hours of medical coverage including work-in clinics [70]. By 2015, more than 60% of GP in Quebec worked in family medicine groups [69]. Created in the 1970s [71], youth centers remain key partners in child-adolescent psychiatry offering specialized regional public services for children and adolescents in difficulty as well as psychosocial, rehabilitation, social integration and placement services [72]. In the consultation-liaison model, the SRP function included close working relationship with these four types of health care providers to reinforce their MH expertise with the aim of improving MHD treatment and increasing numbers of patients with MHD that these clinicians can overall treat.

While research has assessed impacts of the consultation-liaison and other integrated models on patients and GP or other psychosocial clinicians, few studies have explored the perspectives of psychiatrists working as SRP or their managers [25, 58, 73, 74]. Moreover, impacts of the consultation-liaison model and related barriers/facilitators have rarely been considered [25, 58], nor to our knowledge have studies compared implementation of the consultation-liaison model in adult versus child-adolescent psychiatry. A better understanding of SRP perspectives on the consultation-liaison model may help improve future implementation and adaptation of the model leading to better outcomes, while providing guidance to decision-makers involved with future MH reforms, whether in Quebec or elsewhere, especially considering that primary care MH consolidation is a key issue for service improvement. This study may also identify previously unknown barriers or facilitators regarding the implementation of integrated models such as consultation-liaison. Accordingly, this study assessed the main activities of SRP and barriers or facilitators affecting the SRP function from the perspectives of SRP and managers and compared the impacts of SRP in adult and child-adolescent psychiatry in terms of the capacity of Quebec GP, MH-PCT, one-stop MH service teams and youth centers to care for patients with MHD.

## Methods

### Study context

In Canada, health and social services are under provincial jurisdiction and are covered by a universal health insurance system [75]. The Canadian and Quebec health and MH systems have been criticized over the years for inadequate access and continuity of care [76, 77] and for less than optimal consolidation of services within primary care [76, 78]. In the province of Quebec, which accounts for 23% of the Canadian population [79], some 25% of inhabitants are without a “family doctor”, and instead use walk-in clinics as a regular source of care [80, 81]. Wait times to access MH-PCT and psychiatric care are also quite long, varying from several months to more than a year especially for psychiatric care in some Quebec territories [67, 82]. Benchmarks originally set for access to treatment were 1-month for MH-PCT and evaluations within 7-days were promoted for the one-stop MH service teams [65], yet these standards have to be met due to organizational issues and high patient demand [67, 68, 83].

In this context, major recent reforms have been ordered aimed at decreasing the overall number of Quebec health providers while integrating network services; a more recent focus is on the implementation of clinical best practices. In particular, the 2015 health reform [84] created 22 integrated health and social service centers in Quebec, nine of them university-affiliated, merging all public health and social service organizations like hospitals, local community health service centers (including MH-PCT and one-stop MH services) and youth centers into 22 networks (except for 12 university hospitals). Each MH network includes specialized services offered in psychiatric departments of general hospitals or psychiatric hospitals staffed by approximately 1,200 psychiatrists, most (± 90%) working in hospitals [85] and about 44% located in metropolitan Montreal or Quebec, the capital city [86]. Of a total 37 psychiatric departments, 23 serve both adults and children or adolescents through multidisciplinary teams consisting mainly of nurses, psychologists, occupational therapists and social workers. These public MH networks also work in conjunction with GP employed in private family medicine groups on fee-for-service payment schemes to further consolidate MH expertise and better serve patients with MHD.

### Study design

The study used a sequential explanatory mixed-method design [87, 88], with qualitative findings from psychiatric department heads (hereafter: “managers”) on deployment of the consultation-liaison model used to explain and complement the quantitative data from a survey of SRP on their activities and impacts. Mixed methods are particularly useful for understanding the implementation of new programs or reforms [87]. The study was also designed in collaboration with an advisory committee, consisting of representatives from the Quebec Health and Social Services Ministry, the Quebec Association of Psychiatric Physicians, chiefs of psychiatry at MH university institutes and SRP leaders.

### Data collection and instruments

SRP survey data were collected using a self-administered questionnaire and quantitative information from managers using semi-structured interviews. Data collection took place from June 2019 to February 2020 (see Supplementary materials). The questionnaire and interview guide were validated by the advisory committee, and pre-tested by three SRP and three managers. Interviews were conducted by a senior research agent trained and closely monitored by the study researchers. Questionnaire responses were reviewed by the project coordinator to verify that all questions were fully addressed and ensure data quality. The SRP questionnaire required 30 min to complete, while interviews with managers lasted 60 min on average.

The SRP questionnaire included: (1) socio-demographic and professional data (e.g. age, years as SRP); (2) professional activities (e.g. hours per month dedicated to case discussions, telephone availability); (3) profiles of patients discussed or seen (e.g. diagnoses, service use); and (4) perceived impacts of SRP function (e.g. on GP or in MH teams). Manager interviews addressed the organization of SRP (e.g. work integration with MH-PCT or GP) and barriers or facilitators to SRP effectiveness. Manager socio-demographic and professional data (e.g. age, seniority) were also collected through a brief questionnaire (3 min) administered prior to the interviews.

### Recruitment process

First, all departmental managers were contacted to obtain a list of SRP in their respective territories and invited to participate in a semi-structured telephone interview. Invitations to complete the questionnaire were then sent to SRP by email, fax or through departmental secretaries. Up to 12 automatic reminders or emails were sent to non-responders after 2 weeks, or follow-up telephone calls made. Help with recruitment was also sought from the project advisory committee members (December 2019, January 2020). Managers were solicited as often as four times to maximize participation in the study. All participants provided informed consent. The research ethics board of the Douglas MH university institute approved the study protocol.

### Analysis

Regarding quantitative data from SRP questionnaires, the very few missing data found (< 5%) were replaced by the means. First, descriptive statistics including percentages for categorical variables and median or mean values for continuous variables were produced. Second, comparative analyses were conducted to test differences in responses among SRP in adult and child-adolescent psychiatry using the Mann–Whitney U test and Anova. Quantitative data from managers were analyzed using content analysis based on a four-step process [89]: 1) audio-recording of interviews and verbatim transcription; 2) preliminary readings and selection of classification units based on 10% of the verbatim by two research team members working independently, and verification of their high inter-rater reliability by researchers; 3) separation of content of the entire verbatim into units of meaning framed by the interview guide and more broadly, by system, organizational and individual-level barriers or facilitators to deployment of the SRP function; and 4) quantification of qualitative data for weighing the importance of issues discussed by managers. The triangulation process involved: a) summarizing the quantitative data in tables, b) synthesizing the quantitative data in a document, c) validating agreement between results of the SRP survey with information provided by manager, d) and using data from the qualitative investigation to further describe the quantitative results.

## Results

### Sample description (data from questionnaires)

Of 214 SRP identified by managers, 126 participated (response rate = 59%). SRP were mainly female (69%) and 48 years old on average, had worked 16 years as a psychiatrist and six as SRP. Nearly all (90%) were active SRP, 75% working in adult psychiatry, 25% in child-adolescent psychiatry, 65% in general hospital settings versus 35% in psychiatric hospitals, while 42% were in university health regions versus 24%, 19% and 15% in peripheral, remote and intermediary regions, respectively. Of 55 managers invited to the study, 48 participated (response rate = 87%). Managers were mainly female (59%), mean 50 years old with an average of 18 years as a psychiatrist and eight as manager. Moreover, 90% previously or currently worked as SRP, 60% in adult psychiatry and 40% in child-adolescent psychiatry.

### SRP activities (data from the SRP questionnaire and manager interviews)

Table 1 presents the main SRP activities based on the questionnaire. SRP devoted 24.12 h/month on average to their functions, mainly including case discussion meetings, followed by patient consultations with/without clinicians, telephone/video consultations and other. Most SRP (88–96%) performed all these activities, except for patient consultations reported by 56%. Specifically, they provided consultations to one-stop MH service teams (21%), MH-PCT (20%), GP (16%) and youth center patients (10%). Total hours/month reported by SRP and the number of telephone/video consultations with GP and one-stop MH service teams were significantly higher in adult versus child-adolescent psychiatry, whereas the number of SRP consultations with MH-PCT patients was significantly higher in child-adolescent psychiatry than in adult psychiatry.

**Table 1:** Specialist respondent-psychiatrist (SRP) activities per month

	**Total (*n* = 126)**	**Median**^**a**^** (IQR)**^**b**^	**Adult psychiatry (*n* = 94)**	**Median**^**a**^** (IQR)**^**b**^	**Child-adolescent psychiatry (*n* = 32)**	**Median**^**a**^** (IQR)**^**b**^	*P*value*
***a) Total hours for the following activities:***							
**Hours as SRP per month**		24.12 (24.25)		28.20 (25.75)		16.06 (18.53)	0.03
	**N (%)**		**N (%)**		**N (%)**		
**Telephone/video consultations in hours per month (total):** number of SRP participating in the activity and time spent per month	111 (88)	5.00 (13.26)	85 (90)	8.00 (17.00)	26 (81)	5.45 (9.25)	0.02
With general practitioners (GP) in family medicine groups (subtotal)	105 (83)	3.00 (5.00)	81 (86)	4.00 (6.00)	24 (75)	2.50 (3.00)	0.00
With clinicians in mental health (MH) primary care teams (MH-PCT) (subtotal)	60 (48)	1.65 (4.75)	50 (53)	2.00 (5.00)	10 (31)	1.15 (5.50)	0.59
With clinicians in one-stop MH service teams (subtotal)	72 (57)	2.00 (5.00)	57 (61)	2.27 (5.00)	15 (47)	1.50 (1.00)	0.02
With clinicians in youth centers (subtotal)	14 (11)	3.00 (3.25)	0	0	14 (44)	3.00 (3.25)	
**Case discussion meetings (total):** number of SRP participating in the activity and time spent per month	121 (96)	8.00 (9.00)	91 (97)	8.00 (7.00)	30 (94)	5.00 (9.52)	0.06
With GP in family medicine groups (subtotal)	64 (51)	3.00 (2.75)	55 (59)	3.00 (3.00)	9 (28)	4.00 (1.00)	0.96
With clinicians in MH-PCT (subtotal)	86 (68)	3.00 (5.00)	63 (67)	3.00 (5.00)	23 (72)	4.00 (1.00)	0.99
With clinicians in one-stop MH service teams (subtotal)	75 (60)	4.00 (5.00)	62 (66)	4.00 (5.00)	13 (41)	4 .00 (4.50)	0.67
With clinicians in youth centers (subtotal)	14 (11)	2.50 (2.25)	0	0	14 (44)	2.50 (2.25)	
**Consultations with patients (including/not including clinicians) (total):** number of SRP participating in the activity and time spent per month	71 (56)	6.03 (13.00)	55 (59)	6.01 (12.75)	16 (50)	6.03 (15.00)	0.81
With GP in family medicine groups (subtotal)	20 (16)	3.50 (6.00)	17 (18)	2.00 (8.00)	3 (9)	4.00 (0.00)	0.68
With clinicians in MH-PCT (subtotal)	25 (20)	4.00 (6.00)	20 (21)	3.00 (3.00)	5 (16)	5.00 (14.00)	0.04
With clinicians in one-stop MH service teams (subtotal)	26 (21)	3.00 (3.33)	21 (22)	3.00 (3.00)	5 (16)	0.71 (2.29)	0.08
With clinicians in youth centers (subtotal)	12 (10)	2.50 (3.79)	1 (1)	1.00 (0)	11 (34)	3.00 (4.29)	0.60
**Other activities** (e.g. preparation or coordination of clinical services, training, travel)**:** number of SRP participating in the activity and time spent per month	117 (93)	5.00 (7.00)	89 (95)	6.00 (6.00)	28 (88)	4.50 (5.50)	0.29

^a^Median was chosen rather than mean due to the large variation in standard deviation

^b^*IQR* Interquartile range

^*^Mann–Whitney U test *p* value

Table 2 shows the distribution of specific clinical activities per month among SRP. Their time was mainly allocated to GP for pharmaceutical recommendations, to MH-PCT and youth centers for psychosocial and psychotherapeutic recommendations, and to one stop MH service teams for referrals within health service networks. SRP in child-adolescent psychiatry devoted significantly more time toward information sharing on MHD in MH-PCT than those in adult psychiatry.

**Table 2:** Distribution of follow-up clinical activities by specialist respondent-psychiatrists (SRP)

***In percentage per month – distribution of follow-up clinical activities by SRP (100%):***	**Total (*n* = 126)** **Mean (SD)**	**Adult psychiatry (*n* = 94)** **Mean (SD)**	**Child-adolescent psychiatry (*n* = 32)** **Mean (SD)**	*P*value*
**With general practitioners (GP) in family medicine groups with whom SRP worked (N (%)):**	**106 (84)**^**a**^	**81 (86)**^**a**^	**25 (78)**^**a**^	
Information on mental health disorders (MHD)	17 (11)	18 (11)	13 (10)	0.08
Establishment of diagnosis	18 (10)	19 (9)	18 (11)	0.89
Pharmaceutical recommendations	34 (21)	32 (21)	38 (24)	0.39
Psychosocial and psychotherapeutic recommendations	17 (9)	17 (9)	16 (9)	0.49
Orientation in the health service network	12 (8)	11(12)	15 (8)	0.39
Other^b^	2 (12)	3 (14)	0 (1)	0.47
**With clinicians in mental health (MH) primary care teams (MH-PCT) with whom SRP worked (N (%)):**	**93 (74)**^**a**^	**69 (73)**^**a**^	**24 (75)**^**a**^	
Information on MHD	19 (22)	16 (20)	31 (25)	0.04
Establishment of a diagnosis	12 (13)	12 (14)	12 (10)	0.94
Pharmaceutical recommendations	8 (9)	7 (9)	5 (7)	0.47
Psychosocial and psychotherapeutic recommendations	46 (35)	47 (37)	37 (25)	0.34
Orientations in the health service network	14 (17)	17 (13)	15 (11)	0.74
Other^b^	1 (4)	1 (5)	0 (0)	0.51
**With clinicians in one-stop MH service teams with whom SRP worked (N (%)):**	**89 (71)**^**a**^	**68 (72)**^**a**^	**21 (66)**^**a**^	
Information on MHD	13 (13)	12 (13)	18 (15)	0.13
Establishment of a diagnosis	12 (12)	11 (11)	17 (16)	0.15
Pharmaceutical recommendations	10 (12)	12 (12)	8 (10)	0.35
Psychosocial and psychotherapeutic recommendations	19 (18)	18 (19)	20 (15)	0.76
Orientations in the health service network	44 (33)	45 (35)	37 (22)	0.45
Other^b^	2 (9)	2 (10)	0 (0)	0.52
**With clinicians in youth centers with whom SRP worked (N (%)):**	**15 (12)**^**a**^	**1 (1)**^**a,c**^	**14 (44)**^**a**^	
Information on MHD	20 (6)	15 (15)	21 (23)	0.35
Establishment of a diagnosis	22 (8)	14 (14)	23 (12)	0.32
Pharmaceutical recommendations	15 (7)	13 (13)	15 (10)	0.79
Psychosocial and psychotherapeutic recommendations	22 (7)	18 (18)	23 (7)	0.54
Orientations in the health service network	20 (6)	32 (32)	19 (10)	0.03
Other^b^	1 (2)	8 (8)	0 (0)	

^a^Corresponds to the number of participants (n) and percentages (%)

^b^Other: This can include for example administrative activities, discussions on the functioning of team meetings; ongoing feedback, discussions on interview methods or review of clinical records

^c^As there was only one SRP in adult psychiatry who worked with youth centers, we didn’t introduced a comparison

^*^ANOVA t-test *p* value

According to 40% of managers, the total hours covered by SRP was below their cap for 2009, while 10% stated that SRP offered more time than required; the remaining 50% reported rates of SRP coverage in line with government regulations. Questionnaire results indicate that SRP reached 22.0 GP on average, 11.6 clinicians from MH-PCT, 6.2 from one stop MH service teams, and 3.8 from youth centers. SRP in adult psychiatry reached significantly more GP than those in child-adolescent psychiatry (mean 25.6 vs 11.3) and relatively fewer clinicians from adult vs. child-adolescent MH-PCT (9.4 vs 18.0). Managers identified that most network psychiatrists were registered as SRP and provided rotating telephone coverage for brief patient consultations with GP during office hours. However, GP used this service sparingly (median 3 h/month: Table 1).

Managers reported that SRP usually divided their work between sub-networks or MH-PCT and one stop MH service teams or GP family medicine groups. SRP worked with all or most network MH-PCT and one stop MH service teams, whereas half or fewer networks developed partnerships between SRP and GP. MH-PCT consultations were carefully prepared, with psychosocial clinicians sending patient case discussions to SRP in advance; GP were generally not involved in MH-PCT. A maximum of six cases difficult to manage in MH-PCT and requiring SRP assistance were usually discussed per session. SRP provided guidance and recommended treatment based on patient profiles. In one stop MH service teams, SRP mainly supported nurses in evaluating MH demands and orienting patients to appropriate services: whether a return to GP with clinical guidance, referral to the MH-PCT, community services or specialized care to the network hospital link evaluation modules (“module d’évaluation/liaison”). In some networks, SRP or liaison-nurses occasionally met patients for more extended evaluation or provided brief treatment during the wait period for transfer. GP were updated concerning patient profiles and treatments. Regarding family medicine groups in the networks, roughly half of managers noted that the SRP function had not been promoted; only 20% had presented this function to GP through memos, presentations at clinics or to clinic representatives. SRP services had higher priority in larger family medicine groups and those in closer proximity to hospitals employing SRP, in clinics with greater interest in MH and in those where SRP had affiliations or had previously collaborated. Overall, GP were viewed as having little interest in SRP consultation, preferring that SRP consult directly with their patients and providing them with brief orientation. SRP were more successful when targeting “university” family medicine groups, whose training objectives involving medical residents coincided with SRP case discussions. Regarding youth centers, there was usually close collaboration with child-adolescent psychiatry departments, although few managers mentioned that SRP had much contact with them. SRP targeted specialized psychosocial clinicians in youth centers working with patients whose MH conditions were more serious and complex.

### Comfort, motivation, and satisfaction among SRP (questionnaire data, 5-point scales)

SRP comfort and motivation related to their function and capacity to support primary care or youth centers were evaluated as good (3.7/5) (Table 3). SRP evaluated the complexity of their interventions as medium (3.3). They were very satisfied with their degree of influence in decision-making regarding patient referrals and choice of therapeutic interventions (4 +), but less so regarding the clarity of their civil responsibility (3.4). They rated the relevance of their collaboration/consultations as high (4 +), except for GP consultations which were less satisfying (3.6). Satisfaction among SRP regarding the support offered by their organizations was low (2.8) in terms of training activities, feedback on SRP effectiveness or help forthcoming for administrative and logistical tasks. SRP working in child-adolescent psychiatry were significantly more comfortable and motivated around their overall functions than their counterparts in adult psychiatry (3.9 vs 3.6), including the clarity of their role/mandate (4.0 vs 3.7), civil responsibility (3.8 vs 3.3) and their margin of maneuver for accomplishing tasks adequately (4.0 vs 3.5). SRP in child-adolescent psychiatry were also significantly more satisfied with both the administrative and logistical support provided by their organizations (3.4 vs 2.7) and with requests from MH-PCT for their services (4.4 vs 3.5).

**Table 3:** Comfort, motivation and satisfaction related to the function of specialist respondent psychiatrists (SRP)

	**Total (*n* = 126)**	**Adult psychiatry (*n* = 94)**	**Child-adolescent psychiatry (*n* = 32)**	*P*_value*
	**Mean (SD)**^**a**^	**Mean (SD)**^**a**^	**Mean (SD)**^**a**^	
***a) Comfort and motivation related to the SRP function:***	3.70 (0.52)	3.64 (0.52)	3.86 (0.49)	0.04
Level of confidence in SRP capacity to support primary care	3.96 (0.73)	3.90 (0.67)	4.13 (0.87)	0.14
Level of motivation for the function	3.84 (0.83)	3.78 (0.85)	4.03 (0.74)	0.13
Level of complexity regarding interventions to which SRP respond	3.30 (0.58)	3.26 (0.58)	3.44 (0.56)	0.12
**Satisfaction with the SRP role**				
***b) In relation to the function of SRP:***	3.79 (0.65)	3.72 (0.62)	4.00 (0.71)	0.04
Clarity of the role/mandate of SRP	3.83 (0.87)	3.72 (0.86)	4.16 (0.84)	0.01
Clarity of civil responsibility related to SRP function	3.44 (1.07)	3.31 (1.05)	3.81 (1.06)	0.02
Number of hours allocated for adequately carrying out SRP mandate	3.67 (0.96)	3.63 (0.96)	3.81 (0.96)	0.35
Margin of maneuver for accomplishing SRP work adequately	3.62 (1.01)	3.49 (1.03)	4.00 (0.84)	0.01
Degree of influence in choice of appropriate therapeutic interventions – re: cases discussed	4.01 (0.88)	4.01 (0.87)	4.00 (0.91)	0.95
Degree of influence in decision-making regarding patient orientation – re: cases discussed	4.17 (0.82)	4.16 (0.79)	4.22 (0.90)	0.72
***c) In relation to support obtained for the SRP function in their organization:***	2.76 (0.78)	2.72 (0.79)	2.88 (0.74)	0.34
Administrative and logistical support provided by the organization	2.90 (1.22)	2.73 (1.20)	3.38 (1.15)	0.01
Support for training activities on the SRP function	2.45 (1.00)	2.39 (1.07)	2.59 (0.79)	0.33
Feedback on the SRP role aimed at improving SRP functioning and effectiveness	2.70 (1.23)	2.70 (1.31)	2.69 (0.96)	0.95
Optimal support for deployment of the SRP function by psychiatrists from their organization	3.07 (1.01)	3.09 (1.04)	3.03 (0.94)	0.79
Opportunities for exchanges with other SRP	3.10 (1.33)	3.04 (1.33)	3.28 (1.32)	0.38
***d) In relation to collaboration with targeted clinicians:***				
**Adherence to the function with:**	3.63 (1.18)	3.43 (1.58)	3.86 (1.25)	0.40
General practitioners (GP) in family medicine groups	3.34 (0.99)	3.38 (0.96)	3.20 (1.08)	0.43
Clinicians in mental health (MH) primary care teams (MH-PCT)	3.77 (1.30)	3.54 (1.41)	4.37 (0.68)	0.00
Clinicians in one-stop MH service teams	3.93 (1.13)	3.82 (1.23)	4.27 (0.63)	0.10
Clinicians in youth centers	3.48 (1.31)	3.00 (1.81)	3.61 (1.14)	0.36
**Pertinence of consultations/collaborations with:**	3.99 (0.97)	3.96 (1.04)	4.07 (0.91)	0.70
GP in family medicine groups	3.65 (0.97)	3.64 (0.93)	3.68 (1.14)	0.85
Clinicians in MH-PCT	4.04 (1.01)	3.93 (1.08)	4.33 (0.73)	0.07
Clinicians in MH one-stop service teams	4.20 (0.79)	4.19 (0.81)	4.23 (0.75)	0.85
Clinicians in youth centers	4.07 (1.14)	4.11 (1.36)	4.06 (1.05)	0.90
**Stability of professionals with whom SRP interacted (all those mentioned above) in the context of their function as SRP**	3.44 (1.09)	3.41 (1.08)	3.53 (1.16)	0.60

a: 5-point scale: 1 = Very unsatisfied; 2 = Unsatisfied; 3 = Moderately satisfied; 4 = Satisfied; 5 = Very satisfied

^*^ANOVA t-test *p* value

### Profiles of patients discussed or seen in the context of SRP functions (questionnaire data)

Overall, SRP patients were 18–30 years old, single, widowed or divorced and with low income (Table 4). They were mainly affected by common MHD (e.g. depressive disorders), followed by personality and substance-related disorders. Regarding service use, SRP estimated that 67% had a family doctor, and few used MH community organizations, addiction rehabilitation centers or private psychologists. Patient referral to specialized services was justified by severity or complexity of MHD and need for direct and regular psychiatrist intervention.

**Table 4:** Profiles of patients discussed or seen by specialist respondent psychiatrists (SRP)

**Variables**	**Total (*n* = 126)** **Mean (SD)**	**Adult psychiatry (*n* = 94)** **Mean (SD)**	**Child-adolescent psychiatry (*n* = 32)** **Mean (SD)**	*P*_value*
***Patient profile in percentages:***				
***a) Sociodemographic data:***				
Age				
18–30 years	50.99 (32.16)	34.31 (16.82)	100	0.00
31–64 years	37.20 (25.79)	49.86 (15.99)	0	0.00
65 + years	11.81 (12.64)	15.83 (12.27)	0	0.00
Marital status				
Single, widowed or divorced	61.59 (24.21)	52.66 (14.75)	87.81 (27.56)	0.00
Married or with partner	38.41 (24.21)	47.34 (14.75)	12.19 (27.56)	0.00
Income level				
High	15.86 (20.80)	11.63 (8.78)	28.28 (36.04)	0.00
Average	37.27 (18.39)	38.31 (16.49)	34.22 (23.14)	0.27
Low	46.87 (21.13)	50.06 (18.62)	37.50 (25.27)	0.00
***b) Mental health disorders (MHD)***^**a**^***:***	**Mean (SD)**	**Mean (SD)**	**Mean (SD)**	*P*_value*
Common MHD	92.73 (47.54)	91.61 (48.54)	96.04 (45.66)	0.34
Adjustment disorders	20.91 (19.07)	19.98 (18.24)	23.66 (21.38)	0.00
Generalized anxiety	25.17 (16.87)	25.93 (18.46)	22.97 (10.91)	0.39
Depressive disorders	26.60 (17.68)	31.99 (16.65)	10.78 (9.07)	0.00
Attention deficit disorders with/without hyperactivity	20.05 (21.11)	13.72 (15.56)	38.63 (24.36)	0.00
Severe MHD	15.43 (10.63)	18.87 (10.60)	5.33 (6.55)	0.00
Bipolar disorders	7.10 (8.30)	8.86 (8.85)	1.96 (2.38)	0.00
Psychotic disorders (e.g. schizophrenia, delirium)	8.33 (10.41)	10.01 (11.28)	3.37 (4.58)	0.00
Personality disorders	29.50 (20.01)	33.69 (19.36)	17.19 (17.20)	0.00
Substance-related disorders (SRD)	29.53 (27.15)	34.51 (24.56)	14.93 (29.45)	0.00
Alcohol use disorders	13.27 (12.79)	15.90 (10.91)	5.54 (14.83)	0.00
Drug use disorders	16.26 (16.33)	18.60 (15.88)	9.39 (15.92)	.01
**Other** (e.g. eating disorders, pain disorders, intellectual disability)	19.44 (22.64)	15.72 (18.49)	30.36 (29.61)	0.00
**MHD severity**	97.61 (15.30)	97.87 (14.50)	96.87 (17.67)	0.72
MHD 1^e^ episode	32.08 (25.23)	25.74 (17.30)	51.53 (34.02)	0.00
MHD 2^e^ episode	32.89 (18.51)	37.41 (16.53)	19.59 (17.82)	0.00
Chronic MHD	32.44 (34.71)	34.71 (19.77)	25.75 (32.40)	0.06
**c) Other clinical dimensions (not required to equal 100%)**	**Mean (SD)**	**Mean (SD)**	**Mean (SD)**	*P*_value*
High suicide risk	14.33 (14.10)	14.11 (13.94)	15.00 (14.75)	0.75
High risk for aggressiveness	11.79 (13.17)	8.31 (8.64)	22.03 (18.17)	0.00
Housing problems (e.g. homelessness, poor housing)	10.51 (14.38)	11.94 (12.69)	6.31 (18.06)	0.05
Work problems (e.g. loss of employment)	25.42 (22.09)	33.20 (19.62)	2.56 (9.49)	0.00
Problems with activities of daily living	31.02 (21.57)	29.60 (20.40)	35.22 (24.55)	0.20
Chronic physical illnesses	18.75 (17.02)	24.00 (16.50)	3.34 (4.77)	0.00
Social isolation	24.78 (21.34)	29.26 (22.00)	11.63 (12.02)	0.00
***d) Service use (not required to equal 100%):***	**Mean (SD)**	**Mean (SD)**	**Mean (SD)**	*P*_value*
Having a family doctor	66.61 (26.92)	68.78 (25.60)	60.31 (30.02)	0.12
Seeing a private psychologist	10.97 (9.97)	11.36 (9.89)	9.81 (10.28)	0.45
Seeing a clinician in a MH team	33.56 (30.75)	25.23 (25.61)	58.00 (31.94)	0.00
Using one or more MH community organizations	15.49 (15.49)	15.97 (13.76)	14.09 (19.92)	0.55
Receiving services from addiction centers	11.98 (10.56)	13.46 (8.95)	7.64 (13.53)	0.01
Being followed by youth centers	8.76 (19.44)	1.93 (10.55)	28.84 (25.14)	0.00
Qualifying as high users of MH services (e.g. frequent emergency room use for MH reasons > 3 per year, multiple hospitalizations per year)	14.43 (14.99)	14.71 (13.75)	13.60 (18.38)	0.72
***e) Criteria justifying orientation of patients toward MH specialized services*** (**5-point scale: 1 = Never; 2 = Rarely; 3 = Moderately often; 4 = Often; 5 = Very often**)**:**	**Mean (SD)**	**Mean (SD)**	**Mean (SD)**	*P*_value*
MHD too complex or too severe	3.49 (.97)	3.54 (.94)	3.34 (.97)	0.31
Co-occurring MHD and SRD too complex or too severe	2.90 (.86)	3.08 (.79)	2.37 (.85)	0.00
The primary care team has exhausted all intervention avenues, and none have worked	2.82 (.72)	2.80 (.73)	2.89 (.68)	0.55
Needing direct and regular interventions by a psychiatrist	3.13 (.92)	3.06 (.88)	3.06 (1.04)	0.61
Needing services offered by specialized MH services	3.02 (1.00)	3.01 (.97)	3.03 (1.07)	0.80
Patients should be referred to specialized MH services, but refuse for various reasons including fear of stigma	2.00 (.81)	2.25 (.78)	1.73 (.78)	0.00

^a^Patients may have more than one mental disorder – total percentage may exceed 100%

^*^ANOVA t-test *p* value

Compared with patients consulted in primary care or youth centers by SRP in child-adolescent psychiatry, those consulted by SRP in adult psychiatry were generally older, living with a spouse/common-law partner and with low income. They were significantly more affected by depressive, bipolar, psychotic, personality or substance-related disorders, or had experienced a second MHD episode, while less affected by adjustment or attention deficit disorders with/without hyperactivity or other MHD. Work or housing problems, chronic physical illnesses and social isolation were also prevalent, but risk of aggressivity was lower. Moreover, adult patients received more services from addiction rehabilitation centers and were less likely to be followed by MH-PCT or youth centers. SRP in adult psychiatry also referred significantly more patients to specialized services for co-occurring MHD-substance-related disorders, but also encountered more patients who refused referral to specialized services.

### Barriers or facilitators to effectiveness of the SRP function based on manager interviews

Verbatim from the qualitative findings illustrates the main system-level, organizational, and individual barriers/facilitators to the deployment of the SRP function (see Additional file 2). Managers identified slightly more barriers than facilitators, mostly organization-related, followed closely by system-level, then individual factors. Major organizational barriers included instability or frequent MH-PCT staff turnover, but to a lesser degree in one-stop MH service teams (barrier identified by 40% of mangers – hereafter: “% only”); lack of clinician involvement in MH-PCT meetings (30%); insufficient SRP support from hospitals (25%); insufficient coordination mechanisms between one-stop MH service teams, specialized care and SRP (25%); and difficulty managing patients without family doctors in one-stop MH service teams (25%). These patients could have been treated by GP in MH-PCT, had these teams recruited GP or by SRP directly. For more rapid access to specialized care, patients often had to go through emergency departments. Many youth centers were also not prepared organizationally to accommodate SRP (20%). Important organizational facilitators to the SRP function were: having a medical director supportive of SRP in family medicine groups, and interest in MH among psychosocial clinicians in those family medicine groups (30%); working in university family medicine groups with teaching mandates aligned with SRP functions (30%); networks with liaison nurses for care coordination between one-stop MH service teams and specialized care (25%); adequate preparation of case discussions with SRP in MH-PCT (25%); the possibility of referrals to specialized care and direct treatment by the SRP for patients not known to hospitals, optimizing the evaluation process (10%); and in one-stop MH service teams, the provision of brief MH interventions to patients waiting for transfer to either MH-PCT or specialized care (10%).

The main system-level barriers reported were network size and complexity, as when SRP had to travel long distances for team meetings (Web training was not allowed), when the SRP function covered two networks in “borderline” areas or included networks with a large volume of underprivileged patients with complex conditions (60%). Another issue was lack of human resources, especially for psychiatrists working outside the Montreal and Quebec City networks, or where SRP networks were not viewed as a priority but as “icing on the cake” (50%). The inflexibility of the consultation-liaison model particularly in relation to GP needs and modes of remuneration was evaluated as another key barrier (45%). As SRP consultations were organized at regular intervals (e.g. the first Monday of each month), certain GP working at multiple settings were never able to attend. Office space for SRP working in clinics was sometimes problematic. As well, GP paid on a fee-for-service basis generally declined to spend several hours consulting with SRP on “other cases”, preferring brief and immediate responses to their urgent patient needs. Other reported barriers were difficulties distinguishing between MHD appropriate to MH-PCT teams, GP only or specialized care (25%), criteria that could vary according to network expertise and resource availability. Evaluations suggested that SRP spent too much time on MH-PCT consultations (10%) relative to other providers, mostly GP; and that SRP were being highly paid for a service that would otherwise be integrated in their regular practice (10%). Findings identified as main system-level facilitators were: pre-existing relationships with PCT (60%); working in the same hospital as the SRP, more often the case for pediatricians and some MH-PCT (35%); sharing the same electronic medical patient records, which was more characteristic in network hospitals and local community health service centers where MH-PCT and one-stop MH service teams were situated than in family medicine groups that had a separate electronic system (20%); and the optimization of patient treatment (10%). It was thought that the SRP could discuss four cases in the time required by a psychiatrist to complete one patient consultation.

Individual-level barriers related to the reluctance of many GP to treat patients with MHD (40%); discomfort among SRP toward evaluating patients with whom they had not consulted directly, impacting on their civil responsibility (30%); and the delay between GP calls to SRP and their responses which tended to come in the same day, whereas GP often wanted an immediate response (15%). The individual qualities of SRP, their accessibility, sympathetic approach, openness to different practices, knowledge of network resources, and ability to work collaboratively and deal with crises were major facilitators (50%). The ideal SRP would need to be a psychiatrist “champion” to ensure success of this function. Other facilitators were the leadership ability of the head network psychiatrist in supporting the SRP function or ensuring that the SRP function aligned well with main departmental service orientations (30%), and awareness that MH-PCT and one-stop MH service teams consisted of highly respected, senior clinicians.

### Perceived impacts of the SRP function according to SRP self-evaluation (questionnaire data, 5-point scale)

Overall, SRP rated the impacts of their function as moderate (Table 5). They ascribed high impact (4.0 +) only in terms of their ability to perform patient evaluations, particularly through MH-PCT, as well as their coordination and orientation of patients with specialized services, notably for MH-PCT and for one-stop MH services in child-adolescent psychiatry. SRP evaluated their impacts as low (2.0–2.9) on improving the ability of GP to establish a diagnosis, prescribe and orient patients with substance-related disorders toward counseling and psychotherapy. SRP also acknowledged their lack of impacts on the quantity of patients treated by GP or MH-PCT, on timeliness and effectiveness in treating patients referred by youth centers, and on their capacity to increase the supply of MH services in the networks.

**Table 5:** Impact of the specialist respondent psychiatrist (SRP) function on service improvement

**Improvement:**	**Total (*n* = 126)**	**Adult psychiatry (*n* = 94)**	**Child-adolescent psychiatry (*n* = 32)**	*P*_value*
**Of the followed activities:**	**Mean**^a^**(SD)**	**Mean**^a^**(SD)**	**Mean**^a^**(SD)**	
Ability of general practitioners (GP) to establish a diagnosis	2.95 (0.87)	3.01 (0.88)	2.74 (0. 81)	0.15
Pharmacological treatment by GP	3.39 (0.86)	3.47 (0.82)	3.11 0(.93)	0.05
Prescribing and orientation toward counseling and psychotherapy for mental health disorders (MHD) by GP	3.25 (0.91)	3.34 (0.86)	2.96 (1.01)	0.06
Prescribing and orientation toward counseling and psychotherapy for substance-related disorders (SRD) by GP	2.97 (0.87)	3.03 (0.83)	2.76 (0.97)	0.16
**In the ability of these services to evaluate a patient**				
MH primary care teams (MH-PCT)	3.58 (0.98)	3.41 0.97)	4.04 (0.85)	0.00
One-stop MH service teams	3.53 (0.82)	3.53 (0.80)	3.53 (0.90)	0.98
Youth centers	3.74 (0.99)	4.50 (0.70)	3.65 (0.99)	0.26
**In the quality of patient treatment by**				
GP	0	3.25 (0.85)	3.26 (0.94)	0.95
MH-PCT	3.52 (0.98)	3.35 (0.96)	3.93 (0.90)	0.01
**In the quantity of patients treated by**				
GP	2.97 (0.93)	3.01 (0.90)	2.85 (1.04)	0.43
MH-PCT	3.09 (1.14)	2.96 (1.11)	3.44 (1.19)	0.07
**In timeliness and effectiveness of patient treatment requested by**				
One-stop MH service teams	3.41 (0.91)	3.38 (0.96)	3.50 (0.85)	0.64
Youth centers	3.25 (1.15)	2.88 (1.45)	3.44 (0.96)	0.26
**In coordination with MH primary care services**				
GP	3.34 (1.08)	3.30 (1.08)	3.44 (1.12)	0.58
MH-PCT	3.43 (1.08)	3.25 (1.01)	3.89 (1.15)	0.00
One stop MH service teams	3.54 (0.97)	3.39 (0.99)	4.05 (0.70)	0.00
Youth centers	3.46 (0.90)	3.13 (0.83)	3.61 (0.91)	0.21
**In coordination with specialized MH services**				
GP	3.34 (1.10)	3.30 (1.08)	3.44 (1.12)	0.58
MH-PCT	3.65 (1.05)	3.44 (1.07)	4.18 (0.81)	0.00
One stop MH service teams	3.72 (0.88)	3.59 (0.87)	4.16 (0.76)	0.01
Youth centers	3.69 (1.05)	3.13 (1.12)	3.94 (0.93)	0.06
**In the capacity to orient patients toward appropriate services**				
GP	3.40 (1.00)	3.39 (0.98)	3.44 (1.08)	0.80
MH-PCT	3.29 (1.04)	3.20 (1.00)	3.52 (1.12)	0.19
One stop MH service teams	3.44 (0.99)	3.28 (1.02)	4.00 (0.66)	0.00
Youth centers	3.58 (1.04)	3.50 (1.06)	3.61 (0.91)	0.78
**In the capacity to orient patients toward appropriate services for SRD**				
MH-PCT	3.43 (1.08)	3.25 (1.01)	3.89 (1.15)	0.01
One stop MH service teams	3.40 (0.91)	3.39 (0.92)	3.41 (0.87)	0.94
Youth centers	3.22 (0.99)	2.75 (0.70)	3.47 (1.06)	0.10
**Overall impact of the SRP function**	3.22 (0.85)	3.13 0(.81)	3.49 (0.91)	0.04
Increase of MH services offered in the network	2.87 (1.21)	2.77 (1.18)	3.16 (1.27)	0.11
Improvement in patient access to services	3.10 (1.07)	3.06 (1.09)	3.22 (1.03)	0.48
Improvement in the adequacy of services for responding to patient needs	3.48 (1.03)	3.36 (1.03)	3.81 (0.96)	0.03
Improvement in patient health and wellbeing	3.24 (0.96)	3.11 (0.95)	3.62 (0.90)	0.01
Capacity of SRP to support joint follow-up of patient by primary care and specialized MH services	3.37 (1.02)	3.30 (0.93)	3.56 (1.24)	0.20
Better integration and fluidity of services between primary care and specialized MH services	3.32 (0.94)	3.18 (1.10)	3.72 (.1.14)	0.02

a: 5-point scale:1 = No impact; 2 = Weak impact; 3 = Average impact; 4 = High impact; 5 = Very high impact

^*^ANOVA t-test

SRP in child-adolescent psychiatry were significantly more positive than those in adult psychiatry concerning the overall impacts of their function (3.2 vs 2.8) in improving patient health and well-being (3.6 vs 3.1), integrating and promoting fluidity between primary care and specialized services (3.7 vs 3.2), and responding adequately to patient needs for service (3.8 vs 3.4). They were also significantly more confident that their function had improved the capacity of MH-PCT to evaluate (4.0 vs 3.4) and treat (3.9 vs 3.4) patients, the capacity of MH-PCT and one-stop MH service teams to coordinate with primary care (3.9 vs 3.3; 4.1 vs 3.4) and specialized care (4.2 vs 3.4; 4.2 vs 3.6), and coordination between MH-PCT and addiction rehabilitation centers (3.9 vs 3.3). They also viewed themselves as having significantly improved the capacity of one-stop MH service teams to orienting patients toward appropriate services (4.0 vs 3.3). GP improvement in pharmacological treatment was the only area where SRP working in adult psychiatry rated their impact as significantly more positive than that of their colleagues in child-adolescent psychiatry (3.5 vs 3.1).

## Discussion

This study was original in focusing on the main activities of Quebec SRP and their impacts on the capacity of primary care providers (MH-PCT, one-stop MH service and GP) and youth centers to treat and orient patients with MHD. Little has been published on the perceptions of psychiatrists regarding the consultation-liaison model or on the SRP function, specifically in Quebec. Results showed that SRP were implemented sparingly in family medicine groups, while they viewed their overall impacts on the consolidation of primary care and youth centers as moderate. This was also the first study to compare the activities and impacts of SRP working in adult versus child-adolescent psychiatry, with more positive results for the SRP function in the latter.

While previous research estimated the total number of Quebec SRP at 446 [90], roughly half were not active in 2019–20. Our study response rate suggests that fewer than 25% of Quebec psychiatrists worked as active SRP for roughly 1 day a week. Their work focused on supporting either MH-PCT and one-stop MH services, or GP in family medicine groups. Few SRP services were developed in youth centers, and fewer still with GP as compared to MH-PCT and one-stop MH services. Overall, SRP have reached few GP, and the SRP function has not responded as well to their needs as other potential models of integrated MH services. For instance, the use of systematic psychiatric assessment, longitudinal monitoring and care management, as in the collaborative care model, may have better met the needs of GP and patients with MHD, yet these strategies were not included in the consultation-liaison model. As the main providers of MH services for common MHD and gatekeepers to other MH and social services [91, 92], GP might benefit more from the SRP function if they adhered more closely to the collaborative care model. Moreover, the SRP function focused more on consolidating services provided by one-stop MH service teams as crucial for promoting service coordination between MH-PCT and specialized care and for reducing wait time for these services. Developed after 2007 in each Quebec network [67, 93], one-stop MH service teams with the assistance of SRP also aimed to help GP with treatment of patients with MHD, ensuring that only severe and complex cases were transferred to psychiatric care.

Regarding the main activities of SRP, the finding that patient consultation was the second-highest activity reported in terms of median hours per month for over half of SRP suggests that some SRP had incorporated this characteristic of the collaborative care model into their practices [60]. As the 2009 agreement stipulated that SRP could meet patients only exceptionally [66], this change seems to correlate with frequent GP requests that SRP assess their patients directly, bypassing lengthy wait times for accessing one-stop MH service teams or specialized care. The higher total hours per month provided by SRP in adult psychiatry compared with child-adolescent psychiatry can be easily explained by the higher number of primary care clinicians targeting adults for adequate coverage in each network. Both the SRP questionnaire and manager interviews outlined the underutilization of telephone/video availability by SRP between consultations with primary care clinicians or youth centers, and particularly in child-adolescent psychiatry where this service was nonexistent in most networks, mainly due to lack of psychiatrists or the inability of youth centers to coordinate with SRP. In terms of the main SRP activities delivered, the fact that SRP essentially provided GP with medication-related recommendations, MH-PCT and youth centers with recommendations on psychosocial and psychotherapeutic treatments, and one-stop MH service teams with advice on service network orientation was not surprising, as SRP recommendations corresponded to key elements of the mandates under which these service providers operated.

Concerning the comfort level and motivation of SRP, dissatisfaction with the organizational support offered was invoked by both SRP and managers. Formal SRP meetings were rarely held, nor was training or feedback on their activities provided, which may explain the lack of uniformity in their practice. Regarding relationships between SRP and the various teams, their great comfort level with primary care teams but not GP suggests that their specialized expertise was viewed overall as complementary to that offered by psychosocial professionals. Moreover, MH-PCT and one-stop MH service teams consisted largely when created of former MH professionals from specialized services [67], whose positive collaboration with SRP was likely due to previous shared work experiences. Moreover, the greater motivation and comfort of SRP in child-adolescent psychiatry compared with adult SRP may be explained by their longstanding history of consultations in the community, a highly valued aspect of their practice as previously reported [2, 94].

Regarding the profiles of patients met or discussed by SRP, it was not surprising to find a preponderance of patients with low incomes and using few health services, much like patients overall seen in local community health service centers [95, 96]. Moreover, the percentage with a family doctor (67%) reported in the study was slightly inferior to that of the general Quebec population (~ 75%) [97]. SRP patients were also mainly affected by common MHD, corresponding to the profile usually targeted by shared care or collaborative care [29, 38]. As well, the proportion of study patients with personality disorders or substance-related disorders was relatively high. GP and other primary care clinicians usually have limited time to treat and follow up such patients whose relationships with health services are often conflicted [98, 99]. Finally, diagnoses found mainly in child-adolescent psychiatry (adjustment disorders, attention deficit disorders with or without hyperactivity) were those that affected academic performance, social relationship and the safety of children and adolescents [100–103].

Regarding identified barriers/facilitators to the effectiveness of SRP activities, most concerned organizational-level issues. Team instability and frequent staff turnover led to the loss of skills and knowledge hard-won over time [55]. Lack of involvement in some teams may have reflected little interest in the model or doubts about its capacity to improve skills [61]. Regarding the difficulty of managing patients without family doctors, the 2005–2010 Quebec MH Action Plan [65] had recommended two GP per 100,000 inhabitants in MH-PCT, but this target had not been reached. Concerning system-related barriers/facilitators, the availability of appropriate resources [55], payments or incentives [57–59], and preexisting relationships between specialized MH services and other care providers [56] were reported in previous research as key to implementation of the model. Successful deployment of the consultation-liaison model is also impossible without adequate and sufficient human resources, including psychiatrists and GP working in MH-PCT throughout the networks [56, 104]. Not least, preexisting relationships are key to the intensity of collaboration necessary for successful implementation of this model [22]. Regarding individual-level barriers, the reluctance of some GP to embrace the SRP function was reported previously [25]. Discomfort among SRP in assuming joint liability with clinicians for patients not consulted directly by them was another source of discomfort previously reported in a Canadian study [22].

Regarding impacts of the SRP function, the perceived moderate impacts of SRP in this study on the ability of GP to establish a diagnosis, prescribe and orient patients with MHD toward counseling and psychotherapy as well as the quantity of patients treated ran counter to previous results [30, 51]. Considering that the SRP model had little flexibility and seemed mostly unresponsive to GP needs, SRP continued to have difficulty appealing to GP, who too often showed little interest in treating patients with MHD in their current practices, had little time to discuss treatments with SRP and complained that SRP responses to their urgent questions came too late. By contrast, the longer history of collaboration with primary care and youth centers in child-adolescent psychiatry may account for the more positive impacts of SRP there [94]. Another possible explanation is that MH primary care services for children and adolescents in Quebec were less developed than adult services, where better results were attained. Some studies suggest that resource scarcity may encourage interorganizational and inter-professional collaboration [105, 106].

## Limitations

Certain limitations in this study should be noted. First, the study did not investigate the impacts of SRP on patient outcomes. Second, the perspectives of GP and other primary care or youth center clinicians were not investigated. Third, the SRP response rate, while adequate for this type of research with medical doctors, could have been higher. Finally, the results may not be generalizable to other models of integrated MH services in primary care or to diverse MH systems.

## Conclusion

Results suggested that the consultation-liaison model as implemented in Quebec since 2009 differed to some extent from what was initially planned, as evidenced in the time SRP dedicated to direct consultations with patients with or without other clinicians. Results also showed that SRP were generally motivated and comfortable in their function, notwithstanding lack of support from their organizations. Support for SRP needs to be increased in terms of integrating training programs, monitoring meetings, and sharing patient data among providers. Forums for regular professional support among SRP, tailored to their specific needs, are recommended, allowing them to exchange experiences and challenges, increase their comfort/motivation with the SRP function, and feel less isolated in their work. Such meetings could be organized with SRP in adult and child-adolescent psychiatry. Virtual SRP meetings should be encouraged particularly in isolated regions or in geographically extensive areas to promote more efficient collaboration. A standardized provincial guide on best practices for SRP, with particular focus on treating cases of MHD in primary care or specialized services, could be produced. More flexibility toward implementation of the model may also be advisable, as well as better adaptation of the model to territorial characteristics. SRP had a less positive impact on GP in family medicine groups, suggesting the need for increased adaptation of the model to meet GP needs. Perhaps the Quebec model may be more closely aligned with the collaborative care model, based on the generic Wager chronic care model, including the stepped-care approach. Other than modifying GP payment schemes to increase their motivation to collaborate with SRP, the orientations supported by MH reforms need to be better integrated with government directives regarding the consolidation of primary care. Lack of access to a family doctor for all citizens is another major issue that hampers the SRP function. Systemic problems, mainly professional instability, particularly in MH-PCT, long wait times for access to MH-PCT and psychiatric care and the implementation of innovations especially for GP and youth centers should be tackled without delay. Better working conditions including incentives for personnel retention and adaptation of best practices are greatly needed. As well, wider deployment of liaison nurses working closely with SRP and their partners may improve coordination between primary care and specialized MH services. Regarding future research, it would be interesting to compare the perspectives of primary care clinicians with those of SRP and further investigate differences among SRP in terms of their profiles, work characteristics and impacts on primary care MH consolidation.

## Supplementary Information

**Additional file 1.**

**Additional file 2.** Sample quotations for specialist respondent-psychiatrist (SRP) managers: Main barriers (--) and facilitators (++) to implementation of the SRP function.

## Data Availability

The datasets generated and analysed during the current are not publically available, signed confidentiality agreements preventing us from sharing the data, but are available from the corresponding author on reasonable request.

## Abbreviations

GP: General practitioners
MH: Mental health
MHD: Mental health disorders
MH-PCT: Mental health primary care teams
SRP: Specialist respondent-psychiatrists
