# Supplementary material for: Deployment of the consultation-liaison model in adult and child-adolescent psychiatry and its impact on improving mental health treatment
Source: BMC Fam Pract. 2021 Apr 29;22:82. doi: 10.1186/s12875-021-01437-5 (PMC8086343; doi:10.1186/s12875-021-01437-5)
Supplement: Supplementary file 1 — Additional file 1. [file 12875_2021_1437_MOESM1_ESM.docx]

**Questionnaire for specialist respondent psychiatrists (SRP)**

**The function of the specialist respondent psychiatrist (SRP) and shared care: Implementation, practices and impact**

**Funding:** Canadian Institutes of Health Research in collaboration with the Fonds de recherche du Québec – Santé (CIHR-FRQS)

______________________________________________________________________________________

**Principal Investigator:**

Marie-Josée Fleury, PhD., Research Centre, Douglas Mental Health University Institute, McGill University; [flemar@douglas.mcgill.ca](mailto:flemar@douglas.mcgill.ca)

**Co-Investigators:**

Alain Lesage, Université de Montréal

Jacques Tremblay, McGill University

Lambert Farand, Université de Montréal

Pasquale Roberge, Université de Sherbrooke

**Partners:**

Louise Beaudry, Association des médecins psychiatres du Québec

Mimi Israël, Douglas Mental Health University Institute

Jean-Pierre Melun, Institut universitaire en santé mentale de Montréal

Evens Villeneuve, Institut universitaire en santé mentale, Québec

Renée Robichaud, Centre intégré universitaire de santé et de services sociaux, Capitale Nationale

Michel Gervais, Centre intégré universitaire de santé et de services sociaux, Capitale Nationale

André Delorme, Professeur associé, Département de psychiatrie, Université de Montréal

Claude Guimond, Fédération des médecins omnipraticiens du Québec

David Robitaille, Centre intégré de santé et de services sociaux Montérégie-Est, Hôpital Pierre-Boucher

Michel Gilbert, Centre national d’excellence en santé mentale, MSSS

**For further information:**

Armelle Imboua

Research Coordinator

Douglas Mental Health University Institute

6875 LaSalle Boulevard

Montreal (Quebec) Canada H4H 1R3

Telephone: (514) 761-6131, local 3471; Fax: (514) 762-3049

[armelle.imboua@douglas.mcgill.ca](mailto:armelle.imboua@douglas.mcgill.ca)

**Important information to read before completing the questionnaire**

As a specialist respondent psychiatrist (SRP), you are invited to participate in the research project cited on the previous page. This questionnaire is one of the instruments utilized as part of this study, in which the overall objective is to evaluate the implementation, practices and impact of the respondent psychiatrist function since the Quebec mental health reform established in 2005.

The present questionnaire aims to better understand your SRP practice with an adult or youth clientele, and includes 4 sections:

- The first section (A) concern information on the respondent;
- The second section (B) involves an assessment of the SRP function, lines of collaboration and improvements that may be brought to this function;
- The third section (C) aims to develop a portrait of the clientele served;
- Finally, the fourth section (D) concern information on your professional practice as an SRP.

**Period covered by the questionnaire:** the past 12 months (or other as mentioned in the question).

**Types de data:** The questionnaire elicits data regarding your perceptions. We ask you to furnish percentages or approximate numbers according to your experience, and to locate yourself on the assessment scales.

**Duration:** duration of the questionnaire process is about 30 minutes.

**Abbreviations:**

HC: hospital center

CISSS: integrated health and social services center

CIUSSS: integrated university health and social services center

CLSC: local community services centre

Équipe du guichet: one-stop services Teams

MH Teams: Teams of mental health professionals in first line CLSCs, including general practitioners, or not

MH: mental health

MD: mental disorders

SRP: specialist respondent psychiatrist

SUD: substance use disorder

**CONFIDENTIALITY**

All information collected will remain strictly confidential within the limits provided by law. In order to preserve your identity and the confidentiality of the information, you will only be identified by a code number. Further information concerning this point and other ethical considerations are described in the consent form which you are obliged to read, please, sign and return to Armelle Imboua by email (armelle.imboua@douglas.mcgill.ca).

**A. Socio-demographic, and other general informations on the respondent**

* Please note that the information concerning the CISSS / CIUSSS for which you work, as well as your region and your hospital center (CH) will be collected by the research team upon your acceptance to participate in the project.

Note also that you will be asked at the end to indicate how much time it took you to complete the questionnaire

1. What is your date of birth?

________ [Year]

________ [Month]

2. You are?

□ 1 = A man

□ 0 = A woman

3. In what year did you obtain your license to practice as a psychiatrist?

___________

4. In what service of your CH do you mainly work?

*For example: service for psychotic disorders, mood disorders, eating disorders, the emergency dept., etc.

__________________________________

4.1 Would you say that your psychiatric practice is of a “generalist” or “specialist” type?

🞅 1 = Yes, generalist

🞅 0 = Yes, specialist

4.1.1 In what specialization is your practice?

_________________________________________

4.2 Do you exercise a clinical management position?

🞅 1 = Yes

🞅 0 = No

4.2.1 What is this position?

_________________________________________

5. For how long have you practiced as a specialist respondent psychiatrist (SRP)?

*The total number of months and years will correspond to the duration for which you have been a SRP.

________ [Years]

________ [Months]

5.1 Do you work as an SRP on a regular or casual basis?

🞅 1 = Regular

🞅 2 = Casual

5.2 Are you a SRP for youth or adults?

🞅 1 = Youth

🞅 0 = Adults

6. What is your level of confidence in your capacity to support the first line so that they can better manage mental health problems (TM)?

🞅 1 = Very low

🞅 2 = Low

🞅 3 = Neither high nor low

🞅 4 = High

🞅 5 = Very high

7. When you intervene as a SRP, how would you rate the level of complexity regarding the interventions to which you are asked to respond?

🞅 1 = Very low

🞅 2 = Low

🞅 3 = Neither high nor low

🞅 4 = High

🞅 5 = Very high

**B. Assessment and impact of the SRP function**

8. What is your overall level of motivation / satisfaction in your function as SRP?

🞅 1 = Very low

🞅 2 = Low

🞅 3 = Neither high nor low

🞅 4 = High

🞅 5 =Very high

9. In general, how would you evaluate the following aspects?

|  | **Very unsatisfied** | **Unsatisfied** | **Moderately satisfied** | **Satisfied** | **Very satisfied** | **Non applicable** |
| --- | --- | --- | --- | --- | --- | --- |
| 9.1 In line with your general function as SRP | | | | | |  |
| Clarity of your role / mandate |  |  |  |  |  |  |
| Clarity of civil responsibility related to your function |  |  |  |  |  |  |
| Number of hours allocated to you for adequately carrying out your mandate |  |  |  |  |  |  |
| Margin of maneuver as SRP for accomplishing your work adequately |  |  |  |  |  |  |
| Administrative and logistical support provided by your CISSS / CIUSSS |  |  |  |  |  |  |
| Support in terms of training activities on the function of the SRP |  |  |  |  |  |  |
| Feedback on your role as SRP aimed at improving its functioning and effects |  |  |  |  |  |  |
| 9.2 In relation to collaboration with your colleagues in general | | | | | |  |
| Opportunities for exchanges with other SRP |  |  |  |  |  |  |
| Optimal support in the deployment of SRP functions by psychiatrists from your CISSS / CIUSSS |  |  |  |  |  |  |
| Your degree of influence in choice of appropriate therapeutic interventions – for cases discussed |  |  |  |  |  |  |
| Your degree of influence in decision-making regarding patient orientation – for cases discussed |  |  |  |  |  |  |
| Stability of professionals with whom you interact, including general practitioners or clinical Teams (MH, one-stop services or in youth centers) in the context of your function as RSP |  |  |  |  |  |  |
| 9.3 In relation to general practitioners in medical clinics | | | | | |  |
| Adherence to your function  (requests for your services) |  |  |  |  |  |  |
| Pertinence of consultations / collaborations |  |  |  |  |  |  |
| 9.4 In relation to the MH Teams | | | | | |  |
| Adherence to your function  (requests for your services) |  |  |  |  |  |  |
| Pertinence of case discussions / collaborations |  |  |  |  |  |  |
| 9.5 In relation to the one-stop services Teams | | | | | |  |
| Adherence to your function  (requests for your services) |  |  |  |  |  |  |
| Pertinence of case discussions / collaborations |  |  |  |  |  |  |
| 9.6 In relation to clinicians at youth centers | | | | | |  |
| Adherence to your function  (requests for your services) |  |  |  |  |  |  |
| Pertinence of case discussions / collaborations |  |  |  |  |  |  |

10. Considering as a whole your practice as a SRP, what is the impact of your function?

|  | **No impact** | **Weak impact** | **Average Impact** | **High impact** | **Very high impact** | **Non applicable** |
| --- | --- | --- | --- | --- | --- | --- |
| 10.1 On the work of **general practitioners** in the territory? | | | | | |  |
| Improvement of ability to establish a diagnosis |  |  |  |  |  |  |
| Improvement of pharmacological treatment |  |  |  |  |  |  |
| Improvement of prescribing and orientation toward counseling and psychotherapy for MH problems |  |  |  |  |  |  |
| Improvement of prescribing and orientation toward counseling and psychotherapy for substance use disorder (SUD) |  |  |  |  |  |  |
| Improvement in the quality of patient care |  |  |  |  |  |  |
| Improvement in the quantity of patients taken into care |  |  |  |  |  |  |
| Improvement in the capacity to orient patients toward the appropriate service |  |  |  |  |  |  |
| Improvement in coordination with MH Teams / one-stop services Teams |  |  |  |  |  |  |
| Improvement in coordination with specialized services (2^nd^ line) |  |  |  |  |  |  |
| 10.2 On the work of **MH Teams?** | | | | | |  |
| Improvement in the ability to evaluate a patient |  |  |  |  |  |  |
| Improvement in the quality of patient care |  |  |  |  |  |  |
| Improvement in the quantity of patients taken into care |  |  |  |  |  |  |
| Improvement in the capacity to orient patients toward the appropriate service for SUD |  |  |  |  |  |  |
| Improvement in the capacity to orient patients toward the appropriate 1st line services for MH |  |  |  |  |  |  |
| Improvement in coordination with specialized services (2^nd^ line) |  |  |  |  |  |  |
| 10.3 On the work of **one-stop services Teams?** | | | | | |  |
| Improvement in the ability to evaluate a patient |  |  |  |  |  |  |
| Improvement in timeliness and effectiveness in treating patient requests |  |  |  |  |  |  |
| Improvement in the capacity to orient the patient toward the appropriate service for SUD |  |  |  |  |  |  |
| Improvement in coordination / orientation with the 1st line MH Teams |  |  |  |  |  |  |
| Improvement in capacity to orient the patient toward the appropriate 1st line MH service (other than MH Teams) |  |  |  |  |  |  |
| Improvement in coordination with specialized services (2^nd^ line) |  |  |  |  |  |  |
| 10.4 On the work of **clinicians in youth centers?** |  | | | | |  |
| Improvement in the ability to evaluate a patient |  |  |  |  |  |  |
| Improvement in timeliness and effectiveness in treating patient requests |  |  |  |  |  |  |
| Improvement in the capacity to orient patients toward the appropriate service in SUD |  |  |  |  |  |  |
| Improvement in coordination / orientation with the 1st line MH Teams |  |  |  |  |  |  |
| Improvement in capacity to orient the patient toward the appropriate 1st line MH service (other than MH Teams) |  |  |  |  |  |  |
| Improvement in coordination with specialized services (2^nd^ line) |  |  |  |  |  |  |
| **10.5 Overall?** | | | | | |  |
| Increase in MH services offered in the territory |  |  |  |  |  |  |
| Improvement in access to services by patients |  |  |  |  |  |  |
| Improvement in the adequacy of services to respond to patient needs |  |  |  |  |  |  |
| Improvement in the health and wellbeing of patients |  |  |  |  |  |  |
| Capacity of SRP to support joint follow-up of a patient by the first and second lines |  |  |  |  |  |  |
| Better integration and fluidity of services between the 1^st^ and specialized services (2^nd^ line) |  |  |  |  |  |  |

11. In your view, what modifications would help the functioning of SRP in improving services to the population?

|  | **Yes** | **No** |
| --- | --- | --- |
| Sensitize general practitioners to the availability of psychiatrists for telephone consultations |  |  |
| Sensitize general practitioners to the availability of psychiatrists for visits to their clinic for case discussions, patient evaluations or for training |  |  |
| Modify flat fees in order to obtain more flexibility in the available slots  -Increase the ceiling on fee packages for doctors’ offices |  |  |
| Promote harmonization in the provincial scales for the RSP fonction: adoption of a common standards guide for practices, types of tasks, etc. |  |  |
| Be assured of the availability of liaison resources between the SRP, MH Teams and one-stop services Teams as well as with general practitioners or clinicians in youth centers |  |  |
| Review the remuneration and payment system for general practitioners as part of the function of SRP |  |  |
| Specify the legal responsibilities of the SRP |  |  |
| Have access to medico-administrative data on patients seen or discussed as part of the function of the SRP in conjunction with the medical clinics, MH Teams or the one-stop services Teams or the youth centers |  |  |
| Be assured of greater adherence to, and implementation of, the chronic care management model in MH on 1st line as part of the SRP function |  |  |
| Be assured of greater participation of family members in care processes as part of the SRP function |  |  |
| That the SRP may have more direct contact with the patient and be assured of his/her adherence to the treatment plan |  |  |
| Other - Specify |  |  |

12. Within your SRP function, do you engage in promotional activities so that your services will be referred to by more:

|  | **Yes** | **No** |
| --- | --- | --- |
| General practitioners in targeted medical clinics |  |  |
| Clinicians, including general practitioners on MH Teams |  |  |
| Clinicians in one-stop service Teams |  |  |
| Clinicians in youth centers |  |  |

13. At the end of your current mandate, do you intend to pursue your function as an SRP?

🞅 1 = Yes

🞅 0 = No

🞅 97 = Undecided

**C. Portrait of the clientele**

14. What is the overall profile of patients who are the object of discussion (with the general practitioner, MH Teams at either the one-stop services Teams or youth centers), or those you meet as an SRP?

**Response in %, the total must equal 100% when indicated.*

| **14.1 Age** | |
| --- | --- |
| 18-30 years | % |
| 31-64 years | % |
| 65 years and over | % |
| Total | 100% |
| 14.2 Marital status (for MSRP with youth, this refers to parental marital status if they are supporting the young person) | |
| Single, widowed or divorced | % |
| Married or with partner | % |
| Total | 100% |
| **14.3 Income level (**for MSRP with youth, this refers to parental income if they are supporting the young person) | |
| High income | % |
| Average income | % |
| Situation of poverty | % |
| Total | 100% |
| **14.4 Mental health problems of patients** (Percentages in this section are not cumulative – not required to equal 100%) | |
| Adjustment disorders | % |
| Personality disorders | % |
| Depressive disorders | % |
| Bipolar disorders | % |
| Generalized anxiety disorders – DSM-IV (notably generalized anxiety disorders, post-traumatic stress disorder, agoraphobia, obsessive compulsive disorder, etc.) | % |
| Psychotic disorders (ex.: schizophrenia, delirium, etc.) | % |
| Eating disorders | % |
| Attention deficit disorders with or without hyperactivity (ADD/ ADHD) | % |
| Pain disorders / syndromes | % |
| Intellectual disability / Pervasive developmental disorders | % |
| Alcohol use disorders | % |
| Drug use disorders |  |
| Other – specify | % |
| **14.5 Clinical course of patients** (one or more of the problems identified above) | |
| 1st episode of MD | % |
| At least 2 episodes of MD | % |
| Chronic MD | % |
| Total | 100% |
| **14.6 Other clinical dimensions of patients** (Percentages in this section are not cumulative – not required to equal 100%) | |
| High suicide risk | % |
| High risk for aggressivity | % |
| Housing problems (e.g., homelessness, poor housing, etc.) | % |
| Work problems (e.g., loss of employment, etc.) | % |
| Problems in activities of daily living | % |
| Chronic physical illnesses | % |
| Social isolation | % |
| **14.7 Patient services utilization** (Percentages in this section are not cumulative – not required to equal 100%) | |
| Has a family doctor | % |
| Sees a private psychologist | % |
| Sees a clinician on the MH Teams | % |
| Makes significant use of one or more community organizations in MH | % |
| Receives services for problem related to use of drugs or alcohol | % |
| Is followed by youth centres |  |
| May qualify as a high user of MH services (frequent emergency room visits for MH reasons (>3 per year), multiple hospitalizations, etc.) | % |

**D. Description of your professional activities**

15. As a SRP, please indicate how your time would be distributed approximately among the following activities (number of hours per month):

| **Activities** | | **Number of hours / month** |
| --- | --- | --- |
| Telephone consultations when you’re on call (de garde téléphonique) | With general practitioners in medical clinics |  |
|  | With Teams of mental health (MH) professionals in 1^st^ line CLSCs for adults (MH Teams) |  |
|  | With MH Teams at one-stop services (l’équipe du guichet) |  |
|  | With clinicians in youth centers |  |
| Consultations by videoconference, skype, FaceTime, etc., when you’re on call (de garde téléphonique) | With general practitioners in medical clinics |  |
|  | With MH Teams |  |
|  | With MH Teams at one-stop services (one stop-services) |  |
|  | With clinicians in youth centers |  |
| When you’re on call (de garde téléphonique)  On average, how many calls do you receive on the telephone help line in a typical month?  ________________ | |  |
| Telephone consultations when you’re **not** on call | With general practitioners in medical clinics |  |
|  | With Teams MH professionals in 1^st^ line CLSCs for adults (MH Teams) |  |
|  | With MH Teams at one-stop services |  |
|  | With clinicians in youth centers |  |
| Consultations by videoconference, skype, FaceTime, etc., when you’re **not** on call | With general practitioners in medical clinics |  |
|  | With MH Teams |  |
|  | With MH Teams at one-stop services (one stop-services) |  |
|  | With clinicians in youth centers |  |
| Meetings (including group or individual case discussions) | With general practitioners in medical clinics |  |
|  | With MH Teams |  |
|  | With one-stop services Teams |  |
|  | With clinicians in youth centers |  |
| Meetings with patients | With general practitioners in medical clinics |  |
|  | With MH Teams |  |
|  | With one-stop services Teams |  |
|  | With clinicians in youth centers |  |
|  | Without general practitioners, or MH Teams or one-stop services, or clinicians in youth centers, but in the context of your function as SRP |  |
| Visits to medical clinics to support professionals other than physicians (e.g. support to nurses or social workers working with physicians in these clinics) | |  |
| Coordination of clinical services with mental disorder (MD) specialized services | |  |
| Indirect clinical aspects (preparation of meetings, case file notes, etc. – not including travel time) | |  |
| Training offered, not including case discussions or telephone consultations or consultations by videoconference (already included above): to general practitioners, to the MH Teams, to the one-stop services Teams, or to clinicians in youth centers, including information messages and knowledge translation | |  |
| Travel | |  |

15**.** 1 In a typical month, how many hours do you work on average as a SRP?

* Must correspond to the total number of hours inserted in table in question 8

___________ [Number of hours]

15.2. In the course of a typical month, how would you distribute more precisely your clinical activities as a SRP in the context of a meeting/case discussion or consultation with the following professionals?

** Responses are in percentages, so the total for percentages in the table must equal 100*

|  | With **general practitioners**  in medical clinics | With  **MH Teams** | With  **one-stop services Teams** | With **clinicians in youth centers** |
| --- | --- | --- | --- | --- |
| Information on MD | % | % | % | % |
| Suggestions on investigation / establishment of diagnosis | % | % | % | % |
| Pharmaceutical recommendations | % | % | % | % |
| Psychosocial and psychotherapeutic recommendations | % | % | % | % |
| Orientations in the service network | % | % | % | % |
| Other – specify | % | % | % | % |
| SUB-TOTAL | % | % | % | % |
| **TOTAL** | **100%** | | | |

15.3 On average, how many of the clinicians in your assigned* territory within your function as SRP do you reach? (If the choice of response does not apply to your function, notably the choice of youth centres if you work as an SRP for an adult clientele, you may indicate 0%):

*By “assigned territory” we mean the point of care attributed to you in your SRP practice.

a) General practitioners in targeted medical clinics: ________

b) Clinicians including general practitioners in MH Teams: ________

c) Clinicians in one-stop services Teams: ________

d) Clinicians in youth centers: ________

16. In your support activities with general practitioners or the MH Teams or one-stop services Teams or clinicians in youth centers, with what frequency do the following criteria justify the orientation of patients from these entities toward MD specialized services?

|  | **Never** | **Rarely** | **Moderately often** | **Often** | **Very often** |
| --- | --- | --- | --- | --- | --- |
| The patients present with MD too complex or too severe |  |  |  |  |  |
| The patients present with co-occurring MH problems related to drug or alcohol use too complex or too severe |  |  |  |  |  |
| The 1^st^ line Teams has exhausted all avenues of intervention but none has worked |  |  |  |  |  |
| The patient needs direct and regular intervention by a psychiatrist |  |  |  |  |  |
| The patient requires services from a specialized program offered only on MD specialized services (2^nd^ line) |  |  |  |  |  |
| Patients who should be referred to M D specialized services, but who for various reasons, including stigma, prefer to stay on 1^st^ line (refusal of referral to specialized services (2^nd^ line)) |  |  |  |  |  |

17. We are planning to conduct qualitative interviews to further examine elements that facilitate or hinder the implementation and impact of the SRP function. May we contact you again for a short individual interview (approximately 20 minutes)?

🞅 1 = Yes

🞅 0 = No

18. Would you kindly indicate how much time you needed to complete this questionnaire?

______minutes

**We thank you for your participation and for your invaluable collaboration!**

**Interview guide on the function of specialist respondent-psychiatrists (SRP): implementation, practices, and impacts**

**For psychiatric department heads (SRP managers)**

1) How many psychiatrists (including SRP) does your psychiatric department employ (in child psychiatry and in adult services; or overall, if no separate divisions)?

2) How many psychiatrists in your department are registered as SRP?

3) How many hours of service do SRP offer in your department?

a. Do the total hours correspond to the planned for your territory?

i. If fewer hours, tell us why so?

ii. If you used the maximum hours foreseen for the SRP function, was the time allocated to this function sufficient? Please explain your answer.

4) Can you describe for us the organization of SRP, and how requests for SRP services are handled in your territory, both in general and for each of the following groups: family medicine groups; primary mental health teams, one-stop MH service teams, youth centers; specify whether there are distinctions between child psychiatry and adult psychiatry.

5) Can you describe which of the following services are offered by SRP in your territory as well as their modalities of work: for example, consultations with clinicians, individual and group case discussions, whether the patient is present, or not?

6) What mechanisms are used to promote SRP services and their optimal use at the primary care level in your territory?

7) What support strategies do you or your department offer to facilitate SRP services?

8) How are SRP services integrated within the other services offered by your department?

9) Can you describe the main barriers faced by SRP in carrying out their functions?

10) In your opinion, what are the main facilitators that support the SRP role and SRP services?

11) What, in your view, are the main effects of SRP services?
