# Supplementary material for: Deployment of the consultation-liaison model in adult and child-adolescent psychiatry and its impact on improving mental health treatment
Source: BMC Fam Pract. 2021 Apr 29;22:82. doi: 10.1186/s12875-021-01437-5 (PMC8086343; doi:10.1186/s12875-021-01437-5)
Supplement: Supplementary file 2 — Additional file 2. Sample quotations for specialist respondent-psychiatrist (SRP) managers: Main barriers (--) and facilitators (++) to implementation of the SRP function. [file 12875_2021_1437_MOESM2_ESM.docx]

|  |  |
| --- | --- |
| **Additional file 2 –Sample quotations for specialist respondent-psychiatrist (SRP) managers: Main barriers (--) and facilitators (++) to implementation of the SRP function** | |
| **Adult Psychiatry** | **Child-adolescent Psychiatry** |
| ***a) Related to the organization level*** |  |
| *1-Team instability or frequent staff turnover*  (--) ***“****One of the challenges with big teams is the movement of personnel. There are people arriving all the time, people leaving and there are things that are always needing a bit of renewal too.”* *(06404)**  (--) *“Of course, that's a challenge because we've experienced a lot of instability in connection with maternity leave, sick leave, replacement. It is certain that by having a high turnover of staff, the demand is not there.”(01001)*  (--) *“I find that it's always fragile when there are a lot of changes within a team, it's quite a period of adaptation for a team.” (06410)* | *1-Team instability or frequent turnover*  (--) *“It is a team that has renewed itself almost 3 or 4 times in 8 years. It's always a bit hard to start over, to re-explain. All of the things we said 8 years ago and I would say that we can move on to something else, but we have to go over it again.” (16108)*  (--) *“There are teams we gave time to as SRP, such as XX, XY where we went every month. It failed because the team was like a bit dissolved. The team did not rebuild itself enough to be able to make requests.” (06416)*  (++) *“I find that being in a stable team where we finally come to understand our mutual codes and then understand how we work, that helps a lot.” (03007)* |
| *2-Clinical involvement in mental health (MH) primary care team meetings*  (--) *“We really had to have someone who was designated as an instructor to ensure that caregivers were stimulated and then there had to be a number of people involved.*”(12008)  (++) *“I will say the implication of the team. This is the main thing. When the team is very involved in the discussions and appreciates the presence of the psychiatrists.” (06410)* | *2-Clinical involvement in MH primary care team meetings*  (--)*“Sometimes there are cancellations of meetings because there is no case to present or discuss.” (05011)*  (+*+) “It depends on the preparation of the team. If the providers know the patient very well, the answers can be much more specific and richer, then the discussion can really clarify and then help the provider. If the data are more limited, the answers will be more general as well.” (06414)*  (++) *“Longevity is a factor of success. The fact that the team is well established, we understand our mechanisms well.” (02005)* |
| *3-Insufficient support from the hospital*  (--) *“The administrative side, if there are people who take care of requests and appointments and all that, it is major because it is a waste of time.” (14002)*  (++) *“Another issue, sometimes when I have offices, physical spaces, to have a little secretarial support if there are letters to dictate or transcribe, etc.” (05001)* | *3-Insufficient support from the hospital*  (--) *“How it works is that, no, we don't have administrative support on this.” (05011)*  (--) *“I'll stop you right now, there's nothing. Since we have become like a big Integrated University Health and Social Services Centers all that, there are a lot of things that have fallen aside.” (06415)* |
| *4-Insufficiently effective coordination mechanisms with specialized care*  (--) *“The biggest challenge is the articulation with specialized services. Even if there are access criteria with some clarification, sometimes patients were refused in the programs and returned to the one-stop service. (..) The care of patients without a general practitioner (GP) is very difficult indeed. We do an assessment and then after there is no specialized care follow-up required, we find ourselves in complicated situations where we have no one (GP) to refer.” (06404)*  (--) *“Our partners need to link up with three different hospitals. The fact that GP in the community have patients who come from everywhere and not necessarily from our sector, nor psychiatric, neither primary care, complicates matters even more.” (06201)*  (*++) “The same two SRP are the two psychiatrists who work at the links assessment module (in the hospital). So we see the same cases as maybe we have already discussed by telephone or for which we have already questioned for triage.” (06309)* | *4-Insufficiently effective coordination mechanisms with specialized care*  (--) *“I think for the teams in primary and specialized care all the structural changes of the last years were a very big challenge (implementation of current Quebec MH reform). This change led to complicated references to specialized care by the primary care.” (06415)*  (+*+) “What helps a lot is the coordination of services with the emergency room and if necessary with the hospitalization unit, the presence of the same actors everywhere: no break in services, no repetition, no sequential evaluation that does not give much for the patients, I think it adds a lot to the quality.” (06414)*  (+*+) “Basically, when we discuss a file and then say that this file should arrive in child psychiatry (in the one-stop service team), they will finish their report and then they will send it to us, mentioning that it has already been discussed in a meeting. And then our liaison nurse (in the hospital setting) is there at these meetings, she is the one who manages the entire wait list, so when she sees the file go through, she knows that it has already been discussed and what is going on.” (02005)* |
| *5-Family medicine groups or GP:*  *(*--) *“There are some family medicine groups that are provided with the SRP function and others not. It happened a bit naturally, according to the interests of clinicians and GP. So, this gives an incomplete network which is full of holes.” (16301)*  (--) *“There are family doctors still today that categorically refuse to speak to a SRP. It is a requirement that the patient be seen by a psychiatrist, they do not accept anything else.” (030003)*  (++) *“The other success factor is the university family medicine groups, where there are residents, educational structures. It's a lot easier because the bosses are usually less there to deliver, they are also there to teach, so the function of discussing a case is an intrinsic part of their work. Also, when we are there, it is certain that such family medicine groups use us as SRP to teach MH to residents. So it's a bit of a win-win that makes it work much better.” (06101)*  *(++) “I meet the chief of the family medicine department (at the hospital setting) and the person responsible for the family medicine group and then we see if, for example, there are certain more fragile areas in the population or in healthcare, and then we say maybe we should revise the way we treat depression. At that point we will organize training that will meet the needs of clinicians in the family medicine groups.” (11201)* | *5-Family medicine groups or GP or pediatricians:*  (--) *“I find that we have really few links with GP, I have the impression that this is not a formula that is honestly suitable for them. It is suitable for MH teams, the multi-teams with which we work a lot, but GP are never there. GP are only present if there are meetings at the family medicine groups.” (06416)*  (--) *“It is the great loneliness, child psychiatry and pediatricians we do not see each other.* *We do not talk to each other; we do not see each other while we are doing the same job.” (06305)*  (++) *“The links with pediatricians are very fluid, so we do not do half-days with pediatricians, it is on a case-by-case basis, as needed.” (16222)* |
| *6-Youth centers often not organizationally ready to accept SRP in their services*  (--) *“Our youth center - at the moment things are not going well - it is not the professionals but it is the structure, the organization that needs to be supported to open up a place for us.” (11001)* | *6-Youth centers often not organizationally ready to accept SRP in their services*  (--) *“The youth centers in our region have restructured their 2nd level mental health team service offerings and then they are clarifying their request for the SRP, so that we can deploy the service eventually.” (16222)* |
| ***b) Related to the system level*** | |
| *1-Network size and complexity*  (--) *“It is clear that we have a local flavor which is very particular, which is characterized by: homelessness, drug addiction, vulnerable clientele, indigenous, very mobile people who roam from one province to another, students who come from abroad who have little attachment, a lot of isolatio. This has colored the organization of the SRP function in our network.” (06101)*  (--) *“The divisions of primary care and specialized service do not fit. The division of the Integrated Health and Social Services Center does not also consider these historical divisions, so that it is like 3 types of divisions: primary care, specialized services, Integrated Health and Social Services Center.” (161101)*  *(++) “It is a very small environment. I know it's informal, but when I see that a doctor is available I go to see him, we take coffee but talk about serious business. It is this relationship that is the strength of a small system, the relational issues.” (11201)* | *1-Network size and complexity*  (--) *“One of the characteristics is that the territories are very large. So, if we go for example to XX, it takes us about 45 minutes to 1 hour to get to the XX, so, it is certain that it adds an hour to go an hour's journey back to the clinical period of time we do there. So this is a factor that makes it time consuming for us to do the SRP function in our network.” (01003)*  (--) *“These are very multi-ethnic territories, a lot of recent immigrants and refugee populations so there is a lot of work being done using interpreters. This complicated the way services are provided.” (06204)*  (--) *“Historically, in the area, since there was a small pool of psychiatrists and child psychiatrists, there was only one department that was created. Therefore, in child psychiatry, it is Y, but in adult psychiatry, X is in charge of the territory. So we also take care of telephone consultations for young people whose postal code is X. Does it make sense? It complicated things.” (16108)* |
| *2-Lack of human resources*  (--)*“It varies depending on the number of staff in place. If there are fewer GP and they are overwhelmed, it is much more difficult to play the role of SRP.” (11201)*  (--) *"We lack psychiatrists, we run to the right and to the left, so it's not structured enough". (15001)* | *2-Lack of human resources*  (--) *“I see that their team is not sufficient and there are several unfilled positions on their MH primary care team. Of course, that plays out there, if there is no one to take charge, it is sure that it always comes down to the specialized care.” (01003)*  (--) *“The functions of SRP, we always wanted to do it but there are activities that always remain a priority. So every time we don't have enough doctors to cover basic services, the first thing that is diminished is the corollary activities. The limiting factor is really the number of psychiatrists.” (14007)* |
| *3-Inflexibility of the consultation liaison model*  (--) *“GP to my knowledge have no specific remuneration for their patients for whom they discuss with psychiatrists. They have no financial advantage in doing so unlike SRP who go to family medicine groups.” (06101)*  (--) *“For the moment, it remains a challenge to identify family medicine groups who are interested in operating according to the case discussion model. They are interested in receiving a psychiatrist to do psychiatric evaluations, but to bring them more into a model of case discussions and then to support them and help them raise their level in MH and psychiatry, that's something else.” (0604)*  (--) *“There is vagueness between the work of SRP and the work of a psychiatrist consultant as such. These are two functions that complement each other, overlap, and are called upon differently by different people.” (05001)* | *3-Inflexibility of the consultation liaison model*  (--)*“It is as if the role of SRP is to try to support as much as possible the interventions in primary care, but also to put a brake on the requests for consultations towards specialized care. This role of putting a brake creates a bias in the way in which the files are presented, in the expectations that the clinician has when coming to MH one-stop services team, etc.” (03007)*  *(--) “I can see that it is not so suitable for GP. In child psychiatry, we work very little with partner doctors in the context of this function, so there is something else that needs to be put in place. For me this is not yet very clear.” (064160)*  (--) *“Clinicians are not available to do a 3 and a half hour, It would be better if it wasn't a three and a half hour block all the time. Whether it is planned by block of hour or hour and a half because sometimes I think it could even be more effective.” (16222)* |
| *4-Preexisting collaboration; working in the same hospital*  (++) *“We have psychiatrists responding in Local Community Services Centers since 95 in the region. What the SRP function has done is that people have become a little more homogeneous in the different teams.” (13004)*  (++)*“We have well-established cultures of psychiatrists who were part of the MH team even before the last Action Plan, so this was not necessarily a new function. It is a function which has had a new name. (…) There is family medicine group, local community health service center. It is with the latter that we have the greatest relationship because they are in the same building.” (GZ_06410)*  (++) *“Co-location, that the fact that the places are nearby and being able to intervene in the same place, is a factor that is already described in the literature there. So this is important.” (16221)* | *4-Preexisting collaboration; working in the same hospital*  (++) *“There are long-standing agreements where we serve the population in child psychiatry and therefore I think that there is a kind of tradition in fact, between the long-standing primary care and specialized services that actually preceded the establishment of the SRP.” (06415)*  (++) *“What was facilitating is that we already knew each other a little bit. What was also facilitating is that before we were in the same offices. There was a physical closeness.” (11203)*  (++) *“In fact, the pediatricians are right next door. So it's not uncommon for me to get up from my desk up when I have a question for a patient who has something festering in my office, I'll call one in the corridor and vice versa.” (16222)* |
| *5-Other*  (--) *“I think MH teams sometimes express the fact that they have the feeling that they have more serious cases than what is their mandate. That can be a difficulty and then find that it is a bit difficult to refer to the specialized services.”* *(06404)*  (++) *“Some psychiatrists can go to primary care and spend a whole afternoon seeing a patient. I am discussing 4 at the same time, so I find that the model is better suited to the shortage that we are experiencing in our sector for psychiatrists.” (06302)* | *5-Other*  (--)*“There are two files: non-doctor has access to OASIS files, doctors have access and write in the MAIL file. Before seeing a patient, I have to read two files to be sure to have all the information.” (06204)*  (++) *“They send me a fax telling me which patient will be presenting so I can consult the patient's medical file and then see what has already been in his file to already have an idea. So when I go to the table I already like to review at least the file, if it has already been evaluated I have already seen the previous evaluations.” (05011)* |
| ***c) Related to the individual level*** | |
| *1-Reluctance from GP to treat patients with MHD*  (--) *“I don't think that SRP fit what support GP want really. In the region, only half of the patients have a doctor, so those later are overloaded. Psychiatric patients are seen by them as a nuisance, that's a gross generalization of course.” (161101)*  *(--) “Another SRP tried to set up collaboration with a family medicine group, he did some work but, over time, he realized that GP were not really ready to receive our patients. They wanted to use our services without giving us any, so he pulled out.”(06201)* | *1-Reluctance from GP to treat patients with MHD*  *(--) “The main challenges are basically, despite the fact that we try to inform what is our role, what can we do, some GP are reluctant to receive our recommendations and then at the same time, the fact remains that it is their own patients and then that they have not been evaluated directly by us SRP.” (11203)*  *(--)“There are some GP who are not at all interested when they have psychiatric cases. It is said roughly that 1/3 of consultations in general are for MH, thus it is a little surprising.” (15001)* |
| *2-Discomfort of SRP to evaluate patients they did not see directly*  (--)*“One of the issues that emerged was a concern that some SRP may have about giving verbal advice on cases without necessarily having seen the patient.*  *There is always the question of professional liability, of the risk in the event of a dispute or in the event of a complaint, of having to give an opinion without having seen the patient.” (05001)*  (--)*“At the forensic level, you are responsible, so if you haven't seen the patient and you give advice if the patient kills themselves and the family decides to prosecute, you could be at risk.”(06302)* | *2-Discomfort of SRP to evaluate patients they did not see directly*  (--) *“The challenge is also to give your opinion on something that is reported, it is not something that is experienced. So that's for sure it is done with confidence and then it is done with respect, etc., but the fact remains that it is always done by someone there in the sense that the clinical material is the one that is presented for discussion.” (03007)* |
| *3-Individual qualities of SRP*  (+*+) “It takes versatile people, flexible people, because it's not the same way of working as in specialized care, people open to other approaches. We don't always have the same approach, we can learn from people at primary care too and vice versa. It takes a good ability to resolve conflicts because they happen quite often.” (06201)*  (+*+) “It is the personal experience which prevails the collaboration in the care, then the dynamics… The efficiency of a network like that depends on the human factor, taking interest in doing it. The doctors are able to speak to each other; it mostly depends on that.” (12008)*  (*++) “It is also when we know the resources of the community well, that we can inform the GP when it is not necessarily psychiatrist needs but another need, such as an anxiety disorder group, there are some in the community. It is important to know the resources well. Here. The training too.” (14002)* | *3-Individual qualities of SRP*  (*++) “If I continue with the elements of success: the importance of building trust with the teams and I would say like SRP in a relationship that is non-paternalistic to recognize the expertise too of the primary care people.” (06204)*  (+*+) “It takes people who value a lot and who even have the pleasure of sharing in a group. So my recommendation: maintain consistency with flexibility. And then we arrive with flexibility at consistency through mutual respect, and the basic principles of interdisciplinarity, benevolence and the dignity of both the clientele and the members of the team.” (06511)*  *(++) “Longevity, I've been doing this for 10 years anyway, makes it a factor of success.” (02005)* |
| *4-Leadership of psychiatric department heads*  (*++) “I would say that the integration of administrative functions is really a big advantage. When the chief of the psychiatric department is involved in the function of SRP and the medical chief of the ambulatory services is also involved, I think that there is winner too.” (16221)*  (*++) “As for the elements facilitating it is sure that having the support of the chief of psychiatry to do this kind of activity is important, we have to believe in it because the waiting lists of the hospital are always there.” (06201)* | *4-Leadership of psychiatric department heads*  (+*+) “We did not have a chief of the psychiatry department and it was the director of professional services who took over the head of the department and I worked directly with her. I was chief of the child psychiatric services at that time. I had heard about the SRP function and I found that we really needed to work with this model. She supported me a lot in all the things that I put in place.” (15005)*  (*++) “When we need both the institutional support of the sending institution and the institutional support of the institution where the SRP services take place. It's extremely important that we have the institutional support for this position for things to work well. So we are finally talking about the importance of organizational culture, of a culture that wants to move towards collaboration between primary care and specialized care.” (06204)* |
| *5-Other*  *(--)“When GP have a question, often they need the answer right now, not necessarily within a week, if the patient is unstable but not enough to send him to the emergency room. They need an answer which is more grafted to their practice.” (12009)* | *5-Other*  (--) *“In the SRP rules, there is a deadline with which the doctor should be called back in theory. But in the region here like- in the context of the shortage of child psychiatrists- they don't expect me to call back in 20 minutes. Doctors, most of the time, they want to talk to us during the day and they tell parents that they will call them back at night.” (16222)* |

**Code of participants: The first two number indicates the healthcare region of the participant, the third one refers to the Integrated (University) Health and Services Center (when there is only one in the healthcare region, the number is 0), the last two numbers refers to the rank given to the participant in the list of SRP for his or her Integrated (University) Health Services Center or region.*

*Abbreviation: GP: general practitioners; MH: mental health; SRP: specialist respondent psychiatrists.*
